# Tick Bioactive Molecules as Novel Therapeutics: Beyond Vaccine Targets

**DOI:** 10.3389/fcimb.2017.00222

**Published:** 2017-06-06

**Authors:** Kristen E. Murfin, Erol Fikrig

**Affiliations:** ^1^Section of Infectious Disease, Department of Internal Medicine, Yale University School of MedicineNew Haven, CT, United States; ^2^Howard Hughes Medical InstituteChevy Chase, MD, United States; ^3^Department of Microbial Pathogenesis, Yale UniversityNew Haven, CT, United States

**Keywords:** tick, pathogen, microbiota, vector-borne disease, bioactive molecules, therapeutics

Tick-pathogen-host interactions have been closely studied to understand the molecular mechanisms of pathogen transmission for tick-borne diseases, including Lyme disease, babesiosis, spotted fever diseases, and Tick-borne encephalitis, among others. Such studies have yielded insights into disease processes and have identified promising candidates for vaccines against tick-borne diseases (Dai et al., 2009; Schuijt et al., 2011; de la Fuente et al., 2016). In addition to these vaccine targets, the advent of “omics” technologies, such as transcriptomics and proteomics, has opened the doors for discovery of a wide variety of tick bioactive molecules (Francischetti et al., 2005, 2008, 2011; Untalan et al., 2005; Aljamali et al., 2009; Kongsuwan et al., 2010; Karim et al., 2011; Diaz-Martin et al., 2013; Oliveira et al., 2013; Egekwu et al., 2014; Radulovic et al., 2014; Tirloni et al., 2014; Karim and Ribeiro, 2015; Oleaga et al., 2015; Bullard et al., 2016; Kim et al., 2016; Moreira et al., 2017). While some of these bioactive molecules may be applicable for the treatment of tick-borne diseases, many are promising candidates for the treatment of other pathogens or human diseases. Therefore, we propose that careful study of tick bioactive molecules, such as those discovered in “omics” studies, is a promising rich source of novel therapeutics.

## Tick-pathogen interactions

Tick-borne pathogens have a complex lifecycle that involves both a tick and vertebrate host. Within the natural cycle, uninfected ticks acquire pathogens when taking a blood-meal on an infected host. The microbes enter with the blood into the tick's gut. At this point, some pathogens, such as *Anaplasma phagocytophilum* (the causative agent of human granulocytic anaplasmosis), migrate to the salivary glands (Hodzic et al., 1998). Others, such as *Borrelia burgdorferi* (the etiologic agent of Lyme disease) remain in gut (De Silva and Fikrig, 1995). The pathogens are then maintained within the tick organs during molting (De Silva and Fikrig, 1995; Hodzic et al., 1998). Upon the next blood meal, the infectious microbes exit into a vertebrate host with the tick saliva, which is made in the salivary glands (De Silva and Fikrig, 1995; Hodzic et al., 1998). Therefore, microorganisms that remain in the gut through molting must migrate to the salivary glands during the next blood meal.

The complex processes of acquisition and transmission of tick-borne pathogens require specific interactions between the tick, microbe, and host. Indeed, disruption of some tick-pathogen interactions has been shown to decrease transmission (Ramamoorthi et al., 2005; Dai et al., 2009; Zhang et al., 2011; Narasimhan et al., 2014; Coumou et al., 2016). Likewise, vaccination against some tick saliva or salivary gland proteins decreases the ability of the tick to feed on a mammalian host (Gomes et al., 2015; Contreras and de la Fuente, 2016, 2017), which could reduce transmission of pathogens. Therefore, tick proteins that interact with pathogens or facilitate tick feeding have been studied as potential vaccine targets for tick-borne diseases. However, many of these proteins perform biological functions that could also be exploited for therapeutic development.

## Tick bioactive molecules

Perhaps the best-studied source of tick bioactive molecules is tick saliva. Tick saliva includes a cocktail of potent proteins that aid in the feeding of the tick on a mammalian host and improve pathogen transmission from a tick to a mammalian host. These proteins are known to act as anticoagulants, immunosuppressants and immunomodulators, platelet inhibitors, vasodilators, inhibitors of wound healing, and facilitators of tick attachment (Reviewed in Kazimírová and Štibrániová, 2013). Many of these functions have potential uses in the treatment of disease.

For example, coagulation is an important process in many cancers, as it supports tumor growth, angiogenesis, and metastasis (Rickles et al., 2001). Additionally, cancer patients often have complications related to coagulation, such as venous thromboembolisms (Karakatsanis et al., 2016). Treatment of some cancers and cancer complications with anticoagulants has been shown to be effective (Rickles et al., 2001; Karakatsanis et al., 2016). Tick saliva is a rich source of novel anticoagulants that could be exploited for the development of anticoagulants for the treatment of diverse cancers. Indeed, Ixolaris and Amblyomin-X, anticoagulant and antiangionenic proteins from *Amblyomma cajennense*, have shown promising results for the treatment of glioblastoma (Carneiro-Lobo et al., 2009; Barboza et al., 2015), renal cell carcinoma (de Souza et al., 2016), and melanoma (Chudzinski-Tavassi et al., 2010; de Oliveira Ada et al., 2012) in mice. Additionally, complement inhibitors may be useful for disorders of inappropriate complement activation (Baines and Brodsky, 2017) or diseases exacerbated by the complement system, such as cardiovascular disease (Shields et al., 2017). Indeed, *Ornithodoros moubata* Complement Inhibitor (OmCI) has shown promising results in an *in vitro* model of the complement disease paroxysmal nocturnal hemoglobinuria (Kuhn et al., 2016) and a porcine model of myocardial infarction (Pischke et al., 2017). Additional uses for salivary gland proteins include treatment of microbial infections (Cabezas-Cruz et al., 2016; Abraham et al., 2017), autoimmune disease (Sá-Nunes et al., 2009; Soltys et al., 2009), and cardiovascular diseases (Abendschein et al., 2001).

Recently, tick—tick microbiome—pathogen interactions have begun to be studied to understand the implications of the tick microbiome in pathogen transmission. Indeed, perturbing the *Ixodes scapularis* tick microbiome decreases transmission of *B. burgdorferi* (Narasimhan et al., 2014) and increases transmission of *A. phagocytophilum* (Abraham et al., 2017). Study of such interactions can lead to the discovery of novel mechanisms of interaction and potential therapeutics. For example, further work into *A. phagocytophilum*- microbiota interactions determined that *A. phagocytophilum* modulates the tick microbiome during colonization of *I. scapularis*, which facilitates its migration from the tick gut to the salivary glands (Abraham et al., 2017). This occurs through the bacterium inducing expression of the tick gut protein *I. scapularis* antifreeze glycoprotein (IAFGP) (Neelakanta et al., 2010; Abraham et al., 2017), which decreases microbiota biofilms in the tick gut (Abraham et al., 2017). The antibiofilm activity of IAFGP makes it a promising candidate for the treatment of antimicrobial-resistant bacterial pathogens that form biofilms. Indeed, IAFGP expression in flies and mice increases their resistance to bacterial pathogens, such as *Staphylococcus aureus* (Heisig et al., 2014). Additionally, testing in a catheter model demonstrated that IAFGP coatings can inhibit bacterial biofilm formation on medical devices (Heisig et al., 2014). These studies on IAFGP function and potential highlight that other interactions within the tick, such as those between the ticks, pathogens, and microbiomes, are another rich source of bioactive molecules.

## “Omics” studies for the discovery of bioactive molecules

The advent of “omics” technologies, including transcriptomics, proteomics, and genomics, has opened the door for the discovery of new microbial consortium members, host-microbe interactions, and bioactive molecules. Such studies have led to the discovery of many new promising therapeutic candidates, such as animal venom peptides from mollusks (Verdes et al., 2016) and antibiotics from bacteria (Wecke and Mascher, 2011).

The use of proteomic and transcriptomic analyses has uncovered many novel tick-microbe interactions. Additionally, these studies have yielded a multitude of predicted tick bioactive molecules, such as anticoagulants, platelet aggregation inhibitors, vasodilators, antimicrobials, immunosuppressants, immunomodulators, and inhibitors of wound healing (Table 1; Francischetti et al., 2005, 2008, 2011; Untalan et al., 2005; Aljamali et al., 2009; Kongsuwan et al., 2010; Karim et al., 2011; Diaz-Martin et al., 2013; Oliveira et al., 2013; Egekwu et al., 2014; Radulovic et al., 2014; Tirloni et al., 2014; Karim and Ribeiro, 2015; Oleaga et al., 2015; Bullard et al., 2016; Kim et al., 2016; Moreira et al., 2017). These studies have also identified new classes of protein families as well as many proteins of unknown function (Table 1; Francischetti et al., 2005, 2008, 2011; Untalan et al., 2005; Aljamali et al., 2009; Kongsuwan et al., 2010; Karim et al., 2011; Diaz-Martin et al., 2013; Oliveira et al., 2013; Egekwu et al., 2014; Radulovic et al., 2014; Tirloni et al., 2014; Karim and Ribeiro, 2015; Oleaga et al., 2015; Bullard et al., 2016; Kim et al., 2016; Moreira et al., 2017). The vast majority of these bioactive proteins have not been studied in detail, and it is likely that many may be homologs or overlap in function. Therefore, the actual number of discovered bioactive proteins with divergent mechanisms of action is likely less than the total of these studies. However, these studies highlight that there is a vast array of potential bioactive molecules within tick-microbe interactions awaiting further study.

**Table 1:** Proteomic and transcriptomic studies that have predicted novel tick proteins.

**Tick Source^a^**	**Analysis^b^**	**Total identified^c^**	**Bioactive function^d^**	**Protease inhibitor^e^**	**Protease^f^**	**Unknown function^g^**	**Other predicted function^h^**	**Citation^i^**
*Amblyomma americanum* cement cone	Proteomics	33	0	1	4	18	10	Bullard et al., 2016
*Amblyomma americanum* saliva	Transcriptomics and proteomics	895	7	23	18	517	330	Radulovic et al., 2014
*Amblyomma americanum* salivary glands	Transcriptomics and proteomics	5,792	81	98	37	2,608	2,968	Karim and Ribeiro, 2015
*Amblyomma americanum* salivary glands	Transcriptomics	2,002	14	13	2	1,674	299	Aljamali et al., 2009
*Amblyomma maculatum* salivary glands	Transcriptomics and proteomics	15,914	800	379	311	5,389	9,035	Karim et al., 2011
*Amblyomma sculptum* midguts, ovaries and salivary glands	Transcriptomics	27,308	285	79	132	2,312	24,500	Moreira et al., 2017
*Hyalomma marginatum* rufipes	Transcriptomics and proteomics	2,084	35	62	3	722	1,262	Francischetti et al., 2011
*Ornithodoros coriaceus* salivary glands	Transcriptomics and proteomics	726	60	6	13	127	520	Francischetti et al., 2008
*Ornithodoros erraticus* midgut	Proteomics	555	8	0	15	79	453	Oleaga et al., 2015
*Ornithodoros moubata* saliva	Proteomics	193	9	2	7	51	124	Diaz-Martin et al., 2013
*Rhipicephalus (Boophilus) microplus* midgut	Proteomics	142	0	0	3	8	131	Kongsuwan et al., 2010
*Rhipicephalus (Boophilus) microplus* saliva	Proteomics	187	57	29	4	60	35	Tirloni et al., 2014
*Rhipicephalus (Boophilus) microplus* whole ticks	Proteomics	20	0	0	0	12	8	Untalan et al., 2005
*Rhipicephalus sanguineus* saliva	Proteomics	19	2	0	0	4	13	Oliveira et al., 2013
*Ixodes pacificus* salivary glands	Transcriptomics	557	46	21	1	463	26	Francischetti et al., 2005
*Ixodes scapularis* saliva	Proteomics	582	33	43	33	112	361	Kim et al., 2016
*Ixodes scapularis* synganglion	Transcriptomics	41,249	140	0	0	12,660	28,449	Egekwu et al., 2014

a *Source of the tick sample including species name and organ*.

b *Type of analysis performed on the tick sample*.

c *Total number of proteins or transcripts identified by the study*.

d *Total number of predicted proteins that were classified by the study as having a potential bioactive activity, including anticoagulants, platelet aggregation inhibitors, vasodilators, antimicrobials, immunosuppressants, immunomodulators, and inhibitors of wound healing*.

e *Total number of predicted proteins that were classified by the study as potential protease inhibitors. Some protease inhibitors can have bioactive functions of interest, such an immuosuppressant activity*.

f *Total number of predicted proteins that were classified by the study as potential proteases, which can have bioactive functions of interest*.

g *Total number of predicted proteins that were classified by the study as having an unknown function*.

h *Total number of predicted proteins that were classified by the study as having other functions, such as cell junction, energy metabolism, and cytoskeletal functions*.

i *Citation for the study*.

## Development of bioactive molecules into therapeutics

Although “omics” studies have identified a plethora of potential therapeutics, these studies have not led to FDA approval of any novel drugs. In fact, at the time of this publication, no arthropod compound identified by proteomics, transcriptomics, or genomics is in clinical trials in the United States. As mentioned above, this is partially due to lack of follow-up studies on the mechanisms, uses, and optimization of the drug candidates. However, this is likely also due to issues specific to arthropod compounds.

Arthropod compounds often have high cytotoxicity and/or are unstable (Ratcliffe et al., 2014). Therefore, the development of some compounds will require basic research into optimization of the compound, dosage, synthesis methods, and delivery mechanism. For example, Cantharidin, a small molecule toxin from beetles in the Meloidae family, has potent anti-cancer activities and has been shown to be effective against a large variety of cancers (Reviewed in, Deng et al., 2013; Puerto Galvis et al., 2013). However, this compound also has significant toxicity in mammals related to its anticancer activity (Deng et al., 2013; Puerto Galvis et al., 2013; Ratcliffe et al., 2014). Extensive studies have been undertaken to reduce this toxicity through modification of the compound (Deng et al., 2013; Puerto Galvis et al., 2013), alternative production and delivery methods (Chang et al., 2008; Han et al., 2013; Yu and Zhao, 2016), or combination therapies (Wu et al., 2015). These efforts highlight that the resolution of issues, such as toxicity, will require the investment of time and money into basic scientific research for the development process.

Additionally, there are concerns with developing individual compounds from a complex mixture, such as tick saliva. Tick saliva contains a cocktail of potent proteins, and the production of these proteins changes throughout tick feeding (Kim et al., 2016). This suggests that saliva proteins may work synergistically within the context of tick feeding for differing functions or similar functions (e.g., various immunosuppressants could work in concert for greater immunosuppression) at specific time points. Additionally, it is possible that separately encoded proteins or subunits may be necessary for proper function. Therefore, studying individual genes or proteins may miss potential therapeutics. In these cases, it would be necessary to consider co-expression of proteins and/or identify interacting partners within the tick saliva to capture the optimal combinations.

It is worth noting that is some instances the lack of progress toward a viable therapeutic candidate is due to the high cost of drug development rather than a lack of follow-up research. For these compounds, investing in the approval process is not attractive for pharmaceutical companies (Shlaes et al., 2004; Kinter and DeGeorge, 2016). This is the case for many antimicrobials, such as arthropod-derived antimicrobial peptides that target bacterial and fungal pathogens (Ratcliffe et al., 2011).

## Conclusions

Tick-derived bioactive molecules are a promising source of new therapeutics. However, the discovery and development of such compounds is in its infancy. Although some drug candidates have shown promising pre-clinical results, these compounds could fall into the so-called “Valley of Death,” the gap between basic research and translation into treatments. For some therapeutics, this is due to the broad issues common to potential therapeutics: lack of funding for translational research and/or lack of viable pathways for clinical development (Butler, 2008; Collins et al., 2016). However, as discussed in this article, this can also be due to a lack of basic research assessing biological function, potential uses, or optimization of the compound. For tick bioactive compounds to be successfully developed into therapeutics, it will require the investment of basic researchers into the discovery and approval of therapeutic candidates.

## Author contributions

KEM and EF contributed to the writing and editing of the manuscript.

### Conflict of interest statement

The authors declare that the research was conducted in the absence of any commercial or financial relationships that could be construed as a potential conflict of interest.
